# Harbour porpoises respond to climate change

**DOI:** 10.1002/ece3.51

**Published:** 2011-12

**Authors:** Mads Peter Heide-Jørgensen, Maria Iversen, Nynne Hjort Nielsen, Christina Lockyer, Harry Stern, Mads Hvid Ribergaard

**Affiliations:** 1 Greenland Institute of Natural ResourcesBox 570, DK-3900 Nuuk, Greenland; 2 Age Dynamicsc/o Innelvveien 201, Kaldfjord, N-9100 Kvaløysletta, Norway; 3 Polar Science Center, Applied Physics Laboratory, University of Washington1013 NE 40^th^ Street, Seattle WA 98105; 4 Danish Meteorological Institute, Center for Ocean and IceLyngbyvej 100, DK-2100 Copenhagen, Denmark; 5 Greenland Climate Research Center, Greenland Institute of Natural ResourcesBox 570, DK-3900 Nuuk, Greenland

**Keywords:** Climate change, harbour porpoise, west greenland, body condition, Atlantic cod

## Abstract

The effects of climate change on marine ecosystems and in particular on marine top predators are difficult to assess due to, among other things, spatial variability, and lack of clear delineation of marine habitats. The banks of West Greenland are located in a climate sensitive area and are likely to elicit pronounced responses to oceanographic changes in the North Atlantic. The recent increase in sea temperatures on the banks of West Greenland has had cascading effects on sea ice coverage, residency of top predators, and abundance of important prey species like Atlantic cod (*Gadus morhua*). Here, we report on the response of one of the top predators in West Greenland; the harbour porpoise (*Phocoena phocoena*). The porpoises depend on locating high densities of prey species with high nutritive value and they have apparently responded to the general warming on the banks of West Greenland by longer residence times, increased consumption of Atlantic cod resulting in improved body condition in the form of larger fat deposits in blubber, compared to the situation during a cold period in the 1990s. This is one of the few examples of a measurable effect of climate change on a marine mammal population.

## Introduction

The latitudinal gradient along the west coast of Greenland, spanning from 60°N to > 80°N, encompasses the southern tip of Greenland located in the dynamic ocean conditions of the North Atlantic and the northern parts of Greenland located in the more stable high Arctic climate system. Sea temperatures along West Greenland are influenced by large-scale physical processes in the North Atlantic (Myers et al. 2009). The warm, saline Irminger Current, a subsidiary of the North Atlantic Current, meets the south-flowing, low-salinity, cold current of polar origin in the Denmark Strait in East Greenland (Storr–Paulsen et al. 2004; Myers et al. 2007). Both currents flow southward round Cape Farewell and northward along West Greenland, where intense mixing occurs on the productive banks of West Greenland. The strength of the two currents determines the hydrological conditions on the banks, and small changes in the circulation in the North Atlantic have a major impact on the sea temperatures of West Greenland. The presence of warm water in West Greenland plays a crucial role by stimulating production, bringing fish larvae (e.g., Atlantic cod *[Gadus morhua]* and capelin [*Mallotus villosus*]) from spawning areas between East Greenland and Iceland to the banks of West Greenland (Storr–Paulsen et al. 2004; Pedersen et al. 2005). These in turn attract top predators, determine sea ice coverage, and drive the trophic cascade of the marine ecosystem. Since the late 1990s, temperatures of the surface water on the banks of West Greenland have, on average, been above levels measured since the early 1950s (Myers et al. 2009). An extension of the surface water temperature–time series back to 1876 confirms that recent surface water temperatures have been similar or above levels during the period from the mid-1920s to the late 1960s (Ribergaard 2011).

The life histories, behaviors, and feeding patterns of marine mammals are tuned to environmental conditions and associated changes in marine production. One approach for gaining insight into the effects of climate change on marine mammals is to combine long-term monitoring data on population metrics, life history, physiology, or behavior with time series on environmental conditions. The harbour porpoise is a marine endotherm with a high ratio of body surface to body volume. To maintain their high metabolic rate, harbour porpoises depend on locating abundant prey resources at predictable intervals. They are found seasonally where large schools of fish of good nutritive value can be predictably located. Harbour porpoises are generally assumed to occur mainly in shallow coastal waters but they have also recently been found in deep waters far from land (Greenland Institute of Natural Resources unpublished data). In West Greenland, harbour porpoises are hunted for subsistence and the harvest has been increasing since the mid-1990s with a recent average level of 3,500 porpoises per year (http://www.nanoq.gl). The availability of large numbers of harvested porpoises for examination provides a unique opportunity to obtain information on body condition and in turn gain insight into marine ecosystem processes.

## Materials and Methods

### Treatment of samples

**Figure 1:**
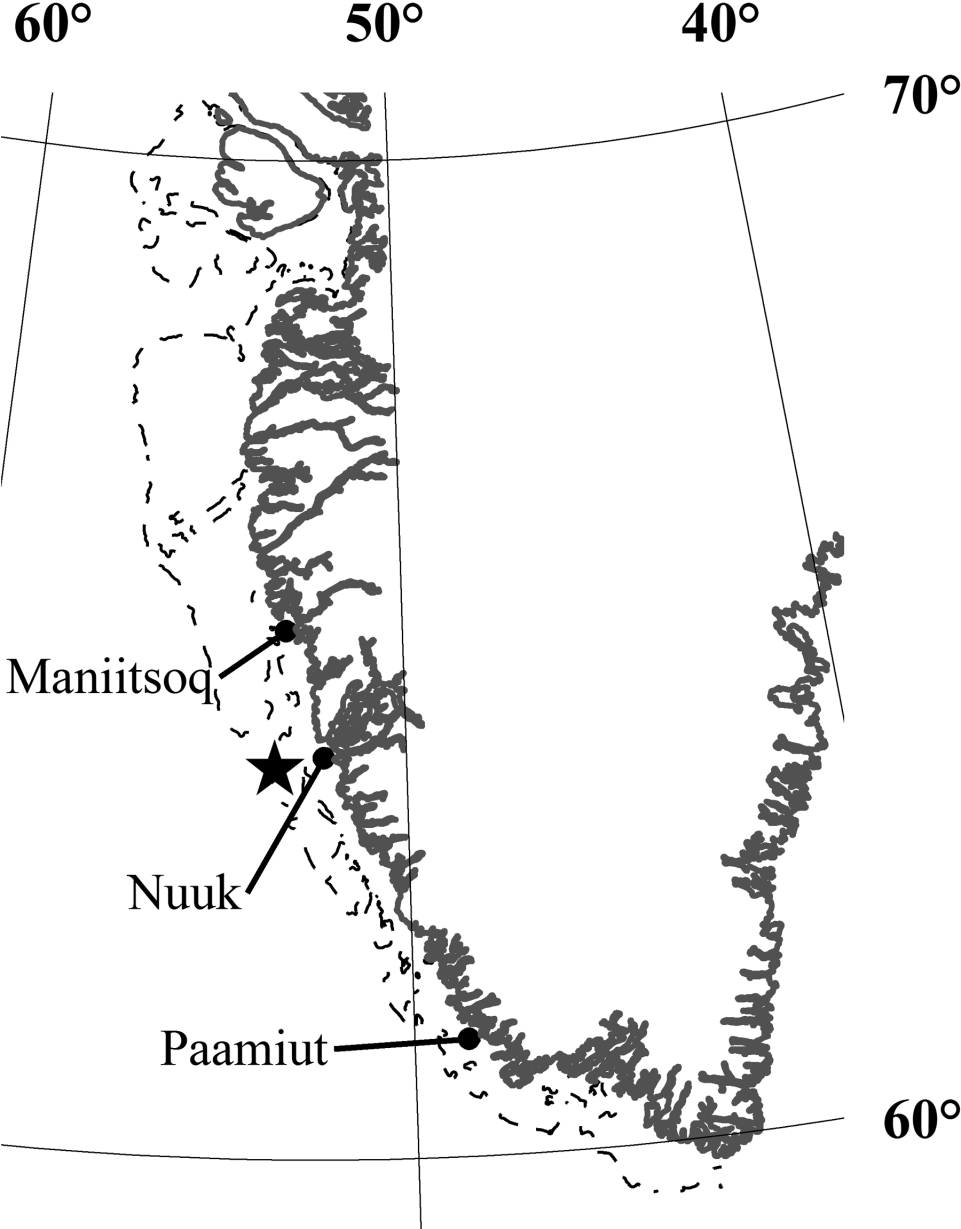
Map of south Greenland showing sampling sites mentioned in the text.

**Figure 2:**
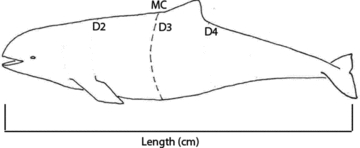
Diagram of selected measurements and sampling sites on harbour porpoises. MC is the maximum circumference, D3–D4 are ventral blubber thicknesses. The length measurement indicated was taken in a direct straight line.

Intact carcasses of fresh harbour porpoises landed in September–October 1995 and 2009 at three towns in West Greenland (Fig. 1) were purchased from local hunters (Lockyer et al. 2001, 2003). The porpoises were frozen whole at the harbour and transported to the laboratory where dissections were conducted. Body length (*L*) was measured in a straight line parallel to the carcass from the tip of the lower jaw to the notch in the tail flukes to the nearest 1 cm. Body mass (*M*) and mass of blubber and skin were measured to the nearest 10 g. Other measures included maximum circumference (*MC*) and blubber thickness (*d*) at three dorsal positions on the porpoises all measured to the nearest 1 cm (Fig. 2). In order to assess the body condition of the harbour porpoises, two standard condition indices were calculated: 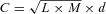 referred to as the LMD index (Ryg et al. 1990) and *C* = *MC/L* (Gales and Renouf 1994) referred to as the MC/L index. Differences between means were tested with student *t*-test and significance was assessed at the 5% level.

The stomach content was washed through 0.5-mm sieves and food items were identified to species or the lowest possible taxon based on sagittal otoliths, skeletal remains, eye lenses, and squid beaks (Härkönen 1986; Campana 2004). The difference in feeding habits between the samples in 1995 and 2009 was tested with a diversity index: 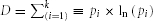 where *k* is the number of prey items and *p* the proportion of observations. Standard error was estimated from a Jackknifed variance estimator. Teeth were removed for age determination following methods previously described (Lockyer et al. 2003). Growth layer groups in both dentine and cementum were examined by two readers and the average was used.

### Sea ice coverage

Sea ice concentration data from satellite passive microwave sensors were obtained from the National Snow and Ice Data Center in Boulder, CO (Cavalieri et al. 1996). The sea ice area in West Greenland was computed as the average over the month of March in the region bounded by 65–70°N and 50–58°W.

### Sea surface temperature

Sea temperatures at depths of between 0 and 40 m taken at a standard station (Fyllas Bank 2: 63°58′N 52°44′W) of the coast of West Greenland each June/July were updated for the years 1950–2010 (Ribergaard 2011). The temperature measurements, corrected for annual variation in order to get the mid-June temperature, were used as a time series in the analysis.

### Cod biomass and porpoise catches

Statistics on the spawning stock biomass of Atlantic cod (*G. morhua*) from 1990 to 2009 were obtained from the International Council for the Exploration of the Sea's Northwestern Working Group Report 2010 (Available from http://www.ices.dk) and statistics on the monthly catches of harbour porpoises from 1993 to 2006 were obtained from the Greenland Home Rule (http://www.nanoq.gl).

## Results

**Figure 3:**
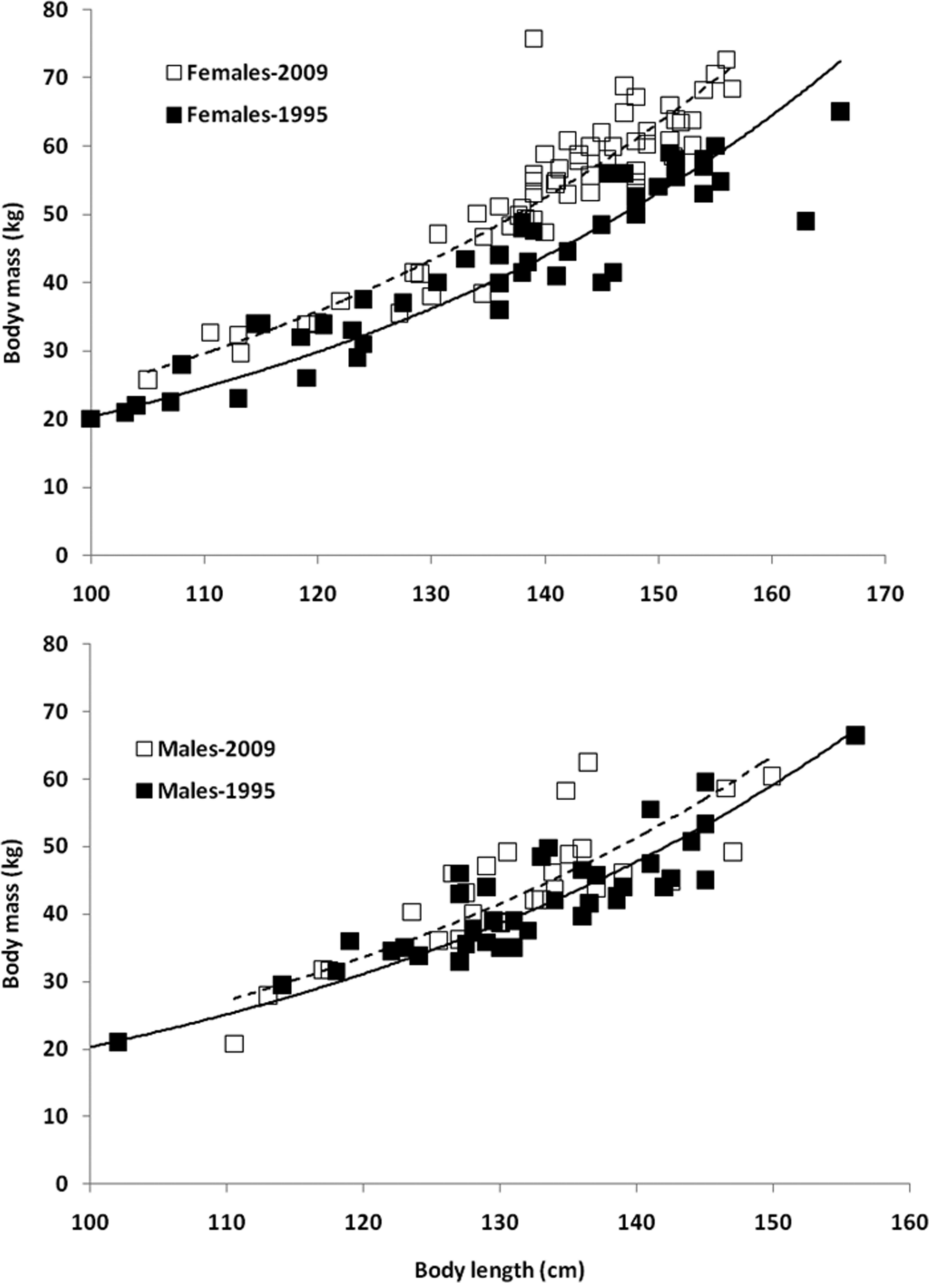
Body mass in relation to body length of female (a) and male (b) harbour porpoises sampled in 1995 (solid line) and 2009 (dashed line) in West Greenland. The data were fitted to an exponential growth model (y = a*e^b^*^x^).

We sampled the catches of harbour porpoises in West Greenland in the fall of 1995 (Lockyer et al. 2001, 2003) and 2009. The female porpoises sampled in 2009 were significantly older than those sampled in 1995 but no difference could be detected for the males. The mean length of those ≥1-year old was similar for the two samples (Table 1). For the comparison of body condition, the data were truncated to the length intervals that were well represented in both sexes, that is, 130 cm ≤ body length ≤ 155 cm for females and 125 cm ≤ body length ≤ 140 cm for males (see Fig. 3). The average body mass for both males and females was significantly larger in 2009, and the blubber mass contributed significantly to the difference (Table 1). The body mass at a given length was larger in 2009 for both sexes (Fig. 3); for a 150-cm female, the difference is about 10 kg or 20%. Comparison of the relationship between body mass and body length showed a significantly (*p* = 0.02) faster increase in body mass for females but the relation is not significantly different for males (Fig. 3, covariance analysis of ln [*M*] on *L*). The LMD body condition index, which included dorsal blubber thickness, showed significant differences between the two sampling years for females but not for males. The MC/L index, which used the circumference of the porpoises was significantly different for both sexes for the two sets of samples. Despite the larger body mass-at-length and improved body condition, no significant difference in age at sexual maturity could be detected.

**Table 1:** Comparison of body condition for harbour porpoises sampled in 1995 (Lockyer et al. 2003) and 2009 in West Greenland (SE in parenthesis). Not all samples were available for all statistics. Data on body condition (see Fig. 1) were truncated to 130 cm ≤ body length ≤ 155 cm for females and 125 cm ≤ body length ≤ 140 cm for males. The LMD condition index is 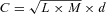 and the MC/L index is *C* = MC/L.^*^ indicate a significant difference at *p* < 0.05, ^*^^*^ indicate *p* < 0.01, and ^*^^*^^*^p < 0.001 between years

	Females 1995 *n* = 55	Females 2009 *n* = 60	Males 1995 *n* = 48	Males 2009 *n* = 29
Mean age > 0 years	3.6^*^ (0.4)	4.9^*^ (0.3)	5.1 (0.6)	5.3 (0.6)
Mean length > 0 years (*L*)	138.1 (2.2)	140.0 (1.5)	133.2 (1.4)	132.0 (1.5)
Mean body mass (*M*)	48.8^*^^*^^*^ (1.3)	56.9^*^^*^^*^ (1.1)	40.9^*^ (1.0)	45.2^*^ (1.6)
Mean age at sexual maturity	3.7^1^ (0.03)	3.5^1^ (0.03)	2.7^2^ (0.03)	3.1^2^ (0.08)
Blubber mass	8.6^*^^*^^*^ (0.17)	10.6^*^^*^^*^ (0.28)	7.6^*^ (0.11)	8.5^*^ (0.45)
LMD condition index D2	1785 (67)^*^^*^	1696 (46)^*^^*^	1696 (46)	1667 (95)
LMD condition index D3	1951 (76)^*^	2207 (67)^*^	1762 (48)	1817 (119)
LMD condition index D4	1967 (74)^*^^*^	2249 (69)^*^^*^	1740 (51)	1768 (94)
MC/L condition index	0.66 (0.005)^*^^*^^*^	0.71 (0.004)^*^^*^^*^	0.68 (0.006)^*^	0.70^*^ (0.008)

1 based on presence of *corpora lutea* and *c. albicantia* in the ovaries;

2 based on combined testes weight > 200g.

Eleven major prey items were detected in the harbour porpoise stomachs in 1995 and this was increased to 23 prey items in 2009 (Table 2). The increase in diversity of the feeding habits between the two samples is significant (*p* < 0.05). Of particular note is that Atlantic cod (*G. morhua*) and Greenland cod (*G. ogaq*) were new prey items that contributed substantially to the diet in 2009. Atlantic cod was detected in 31% and Greenland cod in 19% of the stomachs and the two species were also included in the 59% of the stomachs with unspecific Gadidae. The number of *G.morhua* otoliths in the stomachs ranged between 1 and 49 with an average of 9. Based on the length of the otoliths, the mean length of the ingested Atlantic cod was estimated as 26 cm (SD = 7.3; Härkönen 1986).

**Table 2:** Food preferences of harbor porpoises in West Greenland in 1995 (Lockyer et al. 2003) and 2009 (this study). The percentage of stomachs with each prey item is given. The diversity index was calculated as 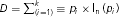 where *k* is the number of prey items and *p* the proportion of observations. Jackknifed standard error is given in parenthesis

Species	2009 (*n* = 88)	1995 (*n* = 77)
Mallotus villosus	78	95
Boregadus saida	53	50
Gadus morhua	31	0
Gadus ogac	19	0
Gadidae	59	0
Lumpenus maculatus	6	0
Sebastes sp.	3	21
Reinhardtius hipploglossoides	3	5
Ammodytidae	2	10
Liparidae	36	7
Lycodes sp.	7	1
Cyclopteridae sp.	1	0
Myctophidae sp.	22	0
Scopelogadus beani	2	0
Salmonidae sp.	2	0
Alepocephalidae sp.	1	0
Cottidae sp.	2	0
Cottuncolidae sp.	9	0
Anarhichas minor	1	0
Squid beaks or eyes	70	25
Pandalus sp.	8	1
Parathemisto libellula	5	13
Thysanoessa sp. and Meganyctiphanes sp.	41	14
Diversity index (SE)	5.4 (0.22)	3.3 (0.16)

**Figure 4:**
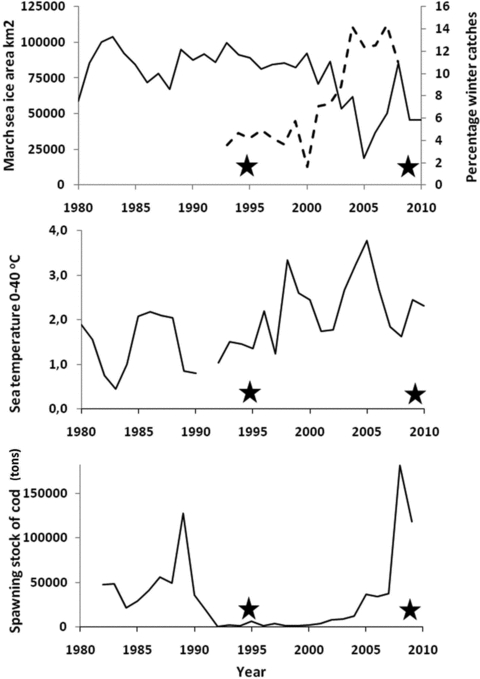
Variations in mid-winter sea ice coverage (a, left axis) along West Greenland with the proportion of annual harbour porpoise catches taken during January–June (a, right axis), sea surface temperature (b) off West Greenland on top of Fylla Bank (0–40 m at Station 2) [5], and estimates of the spawning stock biomass of Atlantic cod off West Greenland from 1982 to 2009 (c). The stars mark the years 1995 and 2009 of the porpoise surveys.

The sea surface temperature on the banks of West Greenland increased significantly (*p* = 0.004) during 1980–2010 with an overall mean of 1.90°C (SD = 0.80) and peak values of 3.23°C and 3.78°C in 2004 and 2005 (Fig. 4). Similarly, winter sea ice coverage declined significantly during 1980–2010 with a simultaneous increase (*p* < 0.001) in the annual percentage of the harbour porpoise catch made during winter months. The extended hunting season indicates longer residence time of porpoises in West Greenland in recent years. Concomitantly, the biomass of the spawning stock of Atlantic cod increased dramatically in 2008 and 2009 as part of a longer-term increasing trend (Fig. 4).

## Discussion

West Greenland experiences extreme climatic fluctuations and these fluctuations have cascading effects on marine life. The most dramatic and economically important effect of the climatic forcing is the periodic irruption of Atlantic cod on the West Greenland banks. These so-called invasions of cod, followed by cold periods where cod are virtually absent from the banks, have been documented since 1700 (Buch et al. 1994, 2005). In the last century, the rise in sea temperature after 1920 coincided with an invasion of cod that led to a dramatic increase in cod abundance in West Greenland, with an international cod fishery initiated 10 years later. After a rapid increase in fishing effort in the 1950s, the cod fishery peaked in the 1960s with catches exceeding 400,000 tons per year. In 1974, quotas were introduced at a time when the catches were declining. In 1982, the biomass was about 10% (Spawning Stock Biomass approximately 100,000 tons) of the biomass in the 1950s (Buch et al. 1994). Catches and fishing activity declined in the 1980s but had a brief recovery in 1988–1990 due apparently to a strong 1984-year class. After 1990, the offshore component of Atlantic cod was virtually absent from the West Greenland banks. Not until 2005 were there signs of a return of the Atlantic cod and simultaneously an increase in sea temperature on the banks. Other great fisheries of subarctic seas have undergone similarly drastic changes but they have proven to be due largely to the effects of overfishing (Fiksen and Slotte 2002). The rise and fall of cod in West Greenland, with the cascading food web consequences, is one of the most pronounced examples of climate forcing on a marine ecosystem.

West Greenland is used by harbour porpoises for seasonal feeding to build up fat deposits as energy reserves. Two factors affect their success on this feeding ground. One is the availability of predictable concentrations of prey of high nutritive value and the other is the seasonal access to ice-free water. Our samples of harbour porpoises represent two contrasting climatic conditions in West Greenland. The summer sea surface temperature has increased dramatically since the late 1990s and it reached a maximum of more than 3.5°C in 2005; over the same period, the maximum extent of winter sea ice coverage retreated considerably and reached its nadir in 2005, with coverage only about 30% of that observed from 1980 to 2000 (Fig. 4). The retreat of the sea ice has occurred mainly along the West Greenland coast, leaving, coastal areas ice free year-round. This is in contrast to the winters between 1979 and 2002 when the >39% sea ice edge consistently reached the coast of West Greenland at 65.4°N (Heide–Jørgensen et al. 2007). Such large-scale changes in ocean conditions have major cascading effects on the trophic structure of the ecosystem and this is clearly demonstrated by both the abundance of cod and the body condition of harbour porpoises.

Harbour porpoises are not adapted to survive in heavy ice conditions and they have difficulty locating and catching prey items in dense sea ice. The percentage of the harbour porpoise catch that was made during the ice season (January–June) tripled between the 1990s and 2004–2008 (Fig. 3). The absence of coastal sea ice in recent years means that harbour porpoises can now be hunted year-round in West Greenland instead of being present only in the ice-free summer season. It is reasonable to infer that the porpoises are present for more of the year nowadays because of the new feeding opportunities associated with ice-free conditions. The greater access for temperate species like the harbour porpoise to the West Greenland feedings banks during winter is furthermore observed by the recovery of Atlantic cod stocks in West Greenland and perhaps also by the influx of several additional temperate fish and krill species that are becoming increasingly important in the diet of harbour porpoises in this region. The diet of the harbour porpoises examined in 2009 was much more diverse than the diet of the sample collected in the same area and at the same time of the year in 1995 (Lockyer et al. 2003). The biggest contributor to the diet in 1995 was capelin (*M. villosus*) and capelin were still present in the 2009 stomachs but the stomachs in 2009 contained twice as many different prey species, and Atlantic and Greenland cod, which were absent in 1995, constituted a major part of the diet. It is infeasible to conduct a full assessment of the diet composition from partly digested material collected from stomachs. Some of the detected remains may be of secondary origin and some species are digested at a faster rate than others (Pierce and Boyle 1991). Nevertheless, cod were the largest of the detected prey items and there is no doubt that the two cod species contributed a major part of the energy intake of the porpoises in 2009.

The longer residence time combined with increased consumption of Atlantic cod has most likely resulted in improved body condition of the porpoises. It is expected that density-dependent regulation would decrease the mean age at sexual maturity when body conditions improve, however, such a change could not be observed. One likely explanation is that the porpoise population, which inhabits West Greenland is already reaching maturity at the earliest possible stage as an age of sexual maturity of about 3 years is the youngest reported for several stocks (Lockyer 2003). The length at sexual maturity is also smaller than in other areas and it is likely that the maturation process cannot be further enhanced by increased per capita food intake.

The ecosystem in West Greenland is relatively simple, with few key species driving the top-level predators and it is therefore not surprising that this area provides some of the first robust evidence of the effects of climate change on the body condition of a marine top predator. The longer feeding season together with the increased cod abundance is the most likely explanation for the pronounced improvement of the body condition of harbour porpoises between 1995 and 2009. Other species of cetaceans are also increasing in abundance in West Greenland (Heide–Jørgensen et al. 2007, in press) or show signs of being affected by the warmer sea temperature there (Laidre et al. 2009), and it can be expected that warming in West Greenland will attract other predator species that are not specialized to endure or exploit Arctic conditions.

The offshore areas along West Greenland provide a unique latitudinal gradient that spans both subarctic and high Arctic marine environments. Due to the complex water currents in the Northwest Atlantic and around the southern tip of Greenland, the banks of West Greenland remain highly susceptible to climatic perturbations and changes in occurrence and phenology of several marine predators including cod, seals, and whales due to climate alterations can easily be detected in West Greenland. Harbour porpoises rely on a continuous supply of prey at predictable concentrations and West Greenland offers an ecosystem with few key prey items for porpoises and where the occurrence of one important prey item (the Atlantic cod) is largely driven by climate forcing (Drinkwater et al. 2010). At the same time, reduced sea ice conditions allow for extended residence times at the Greenland banks where the porpoises can prey intensively on the abundant resources of cod. Due mainly to spatial variability and lack of clear delineation of marine habitats, it is usually hard to detect such direct links between climate forcing and the body conditions of a top predator. It is therefore concluded that monitoring of predator condition is a useful approach for gaining insight into ecosystem changes that are otherwise hard to discern in marine habitats.
